# Identification and analysis of isoflavone reductase gene family in *Gossypium hirsutum* L.

**DOI:** 10.1038/s41598-023-32213-3

**Published:** 2023-04-07

**Authors:** Yanting Gui, Guozhan Fu, Xuelin Li, Yinghao Dai

**Affiliations:** College of Agronomy, Henan University of Science and Technology, Luoyang, 471023 People’s Republic of China

**Keywords:** Genetics, Plant sciences

## Abstract

Isoflavone reductase (*IFR*) is a key enzyme controlling isoflavone synthesis and widely involved in response to various stresses. In this study, the *IFR* genes in four *Gossypium* species and other 7 species were identified and analyzed in the whole genome, and the physicochemical properties, gene structures, *cis*-acting elements, chromosomal locations, collinearity relationships and expression patterns of *IFR* genes were systematically analyzed. 28, 28, 14 and 15 *IFR* genes were identified in *Gossypium hirsutum*, *Gossypium barbadense*, *Gossypium arboreum* and *Gossypium raimondii*, respectively, which were divided into five clades according to the evolutionary tree and gene structure. Collinear analysis showed that segmental duplication and whole genome duplication were the main driving forces in the process of evolution, and most genes underwent pure selection. Gene structure analysis showed that *IFR* gene family was relatively conserved. *Cis*-element analysis of promoter showed that most *GhIFR* genes contain *cis*-elements related to abiotic stresses and plant hormones. Analysis of *GhIFR* gene expression under different stresses showed that *GhIFR* genes were involved in the response to drought, salt, heat and cold stresses through corresponding network mechanisms, especially *GhIFR9A*. Phenotypic analysis after silencing *GhIFR9A* gene by VIGS was shown that *GhIFR9A* gene was involved in the response to salt stress. This study laid a foundation for the subsequent functional study of cotton *IFR* genes.

## Introduction

Cotton is an important cash crop, and a pioneer crop in saline-alkali land. It has certain saline-alkali resistance and is an important model crop for us to study saline-alkali stress^1^. However, it can also be affected by various abiotic stresses during growth and development, such as salt, alkali, drought, low temperature, heat stress and so on. Plants can produce many secondary metabolites to eliminate harmful reactive oxygen species (ROS), thus promoting growth and development and resisting various biotic and abiotic stresses^2^. As an important secondary metabolite, isoflavones play a vital role in plant growth and development. Isoflavones belong to estrogen-like active substances, which can be classified as phytoestrogens^3^. Isoflavones are a subclass of flavonoids, and they are the major phytoestrogens naturally found in plants, such as peanuts, chickpeas, green peas, and alfalfa, and they are mainly produced in the leguminous plants. Previous studies had focused on the ability of flavonoids to relieve stress, mainly by increasing a wide range of biological activities including antioxidant and antifungal properties^4^. There were also investigations of flavonoids improving cotton's development^5^. In addition, flavonoid biosynthesis pathways regulate axillary bud growth by promoting the transport of auxin in upland cotton^6^. More recently, it was found that flavonoids were involved in response to low phosphorus stress^7^. However, the alleviation of abiotic stress in cotton by isoflavones has not been reported.

Isoflavone reductase (*IFR*) is a key enzyme in isoflavone synthesis pathway, which can control the synthesis of isoflavone in plants. At the same time, *IFR* is also involved in lignin synthesis to resist the stresses^8^. It was found that *IFR* gene was also involved in response to biotic and abiotic stresses. In soybeans, ABA treatment induces the expression of isoflavone reductase to resist ABA stress through the synthesis of secondary metabolites^9^. Under waterlogging stress, the expression of isoflavone reductase related genes was significantly up-regulated, which may play an important role in waterlogging stress^10^.

In addition, some studies have elucidated the function of *IFR* gene. In rice, overexpression of isoflavone reductase-like gene (*OsIRL*) can enhance tolerance to ROS stress^11^. Overexpression of *GmIFR* in soybeans can increase soybean resistance to *Phytophthora sojae*^12^. *IRL* gene in rice plays an important role in resisting heat stress^13^. *IFR* gene is also involved in the biosynthesis of tobacco alkaloids to resist various stresses^14^. However, it has not been known whether *IFR* gene is involved in abiotic stress in cotton.

In recent years, the genome sequencing of two diploid cotton *Gossypium arboreum* (*G. arboreum*)^15^ and *Gossypium raimondii* (*G. raimondii*)^16^ and two allotetraploid cotton *Gossypium hirsutum* (*G. hirsutum*)^17,18^ and *Gossypium barbadense* (*G. barbadense*)^19^ has been completed, which provides strong support for our research on the evolution of *IFR* gene family and the potential function of *IFR* genes.

To investigate the evolution and potential functions of the *IFR* gene family, we analyzed the *IFR* phylogenetic tree, gene structure, chromosomal position, collinearity relationship, and promoter *cis-*acting elements. In addition, the heat map of *GhIFR* gene under different stresses was analyzed. A key gene that is highly expressed in salt stress, *GhIFR9A*, was confirmed to positively regulate salt stress by VIGs assay. This study laid a foundation for further research on the function of *IFR* gene and its response to abiotic stresses.

## Results

### Identification of IFR family genes

The HiddenMarkov model of NmrA was used as a query file, and genes with conserved domain of NmrA were selected as candidate genes of *IFR* gene family, and Pfam database was used for further analysis based on the number PF05368. After further screening *IFR* genes by using CD search, we finally identified 28, 28, 14 and 15 *IFR* genes in *G. hirsutum*, *G. barbadense*, *G. arboreum* and *G. raimondii*, respectively. Similarly, we characterized *IFR* genes in other 7 species including *A. thaliana*, *G. max*, *P. trichocarpa*, *O. sativa*, *T. cacao*, *V. vinifera* and *Z. mays*, and 7, 36, 10, 7, 6, 7 and 4 *IFR* genes were identified. To facilitate subsequent identification of *IFR* genes, we renamed them according to the location of chromosomes (Supplementary Table S1). Among them, *IFR* genes in *G. hirsutum* were named *GhIFR1A*-*GhIFR14A* and *GhIFR1D*-*GhIFR14D*. The *IFR* genes in *G. barbadense* were named as *GbIFR1A*-*GbIFR13A* and *GbIFR1D*-*GbIFR15D*. The *IFR* gene in *G. arboreum* was renamed as *GaIFR1*-*GaIFR14*. The *IFR* gene in *G. raimondii* was renamed as *GrIFR1*-*GrIFR15*. The combined number of *IFR* genes of *G. arboreum* and *G. raimondii* was 29, almost equal to that of *G. hirsutum* or *G. barbadense*. We also analyzed the physical properties of *IFR* gene in *G. hirsutum* (Table 1). The protein length ranged from 270 amino acids (*GhIFR8A*) to 747 amino acids (*GhIFR2A*), and the molecular weight ranged from 30.343 kDa (*GhIFR8A*) to 81.643 kDa (*GhIFR2A*). The minimum isoelectric point (pI) was 5.005 (*GhIFR14D*), the maximum was 10.148 (*GhIFR2A*), and the average pI was 7.105. The grand average of hydropathy was negative, indicating that most GhIFR proteins were hydrophilic proteins.

**Table 1:** Physical properties of the *GhIFR* genes.

Gene ID	New gene ID	Protein length (aa)	Molecular weight (kDa)	Charge	Isoelectric point	Grand average of hydropathy
*Gh_A01G0933*	*GhIFR1A*	308	33.979	− 1.5	6.059	− 0.086
*Gh_A03G0704*	*GhIFR2A*	747	81.643	31.5	10.148	− 0.580
*Gh_A03G1719*	*GhIFR3A*	307	34.404	− 5.5	5.239	− 0.088
*Gh_A03G1849*	*GhIFR4A*	398	44.305	8.5	8.387	− 0.124
*Gh_A04G1088*	*GhIFR5A*	318	36.102	− 4.5	5.460	− 0.084
*Gh_A05G0995*	*GhIFR6A*	592	65.231	17.0	9.464	− 0.392
*Gh_A06G1170*	*GhIFR7A*	583	64.165	9.0	8.999	− 0.266
*Gh_A06G1714*	*GhIFR8A*	270	30.343	− 3.0	5.692	− 0.183
*Gh_A08G1366*	*GhIFR9A*	313	35.173	− 3.0	5.885	− 0.091
*Gh_A08G1368*	*GhIFR10A*	312	34.879	− 0.5	6.427	− 0.129
*Gh_A10G2274*	*GhIFR11A*	411	45.170	5.5	8.446	− 0.053
*Gh_A12G0592*	*GhIFR12A*	308	34.679	− 4.5	5.278	− 0.140
*Gh_A12G1558*	*GhIFR13A*	351	39.022	− 3.0	5.891	− 0.188
*Gh_A12G2406*	*GhIFR14A*	359	39.750	− 5.5	5.119	− 0.174
*Gh_D01G0977*	*GhIFR1D*	308	33.855	− 3.0	5.691	− 0.056
*Gh_D02G0949*	*GhIFR2D*	746	81.404	29.0	10.098	− 0.596
*Gh_D02G2151*	*GhIFR3D*	306	34.213	− 1.5	6.171	− 0.094
*Gh_D02G2288*	*GhIFR4D*	398	44.231	8.5	8.387	− 0.120
*Gh_D05G1113*	*GhIFR5D*	595	65.548	17.0	9.461	− 0.402
*Gh_D06G1457*	*GhIFR6D*	615	67.621	11.0	9.114	− 0.261
*Gh_D06G2263*	*GhIFR7D*	322	36.066	1.5	6.891	− 0.127
*Gh_D08G1661*	*GhIFR8D*	313	35.200	− 2.0	6.111	− 0.105
*Gh_D08G1662*	*GhIFR9D*	312	34.865	− 0.5	6.427	− 0.126
*Gh_D10G1660*	*GhIFR10D*	411	45.205	4.5	8.252	− 0.051
*Gh_D11G1380*	*GhIFR11D*	412	45.173	10.0	9.053	− 0.107
*Gh_D12G0604*	*GhIFR12D*	306	34.325	− 4.0	5.457	− 0.065
*Gh_D12G1686*	*GhIFR13D*	351	39.077	− 1.0	6.318	− 0.209
*Gh_D12G2642*	*GhIFR14D*	359	39.879	− 7.0	5.005	− 0.184

### Phylogenetic analysis of *IFR* gene family

**Figure 1:**
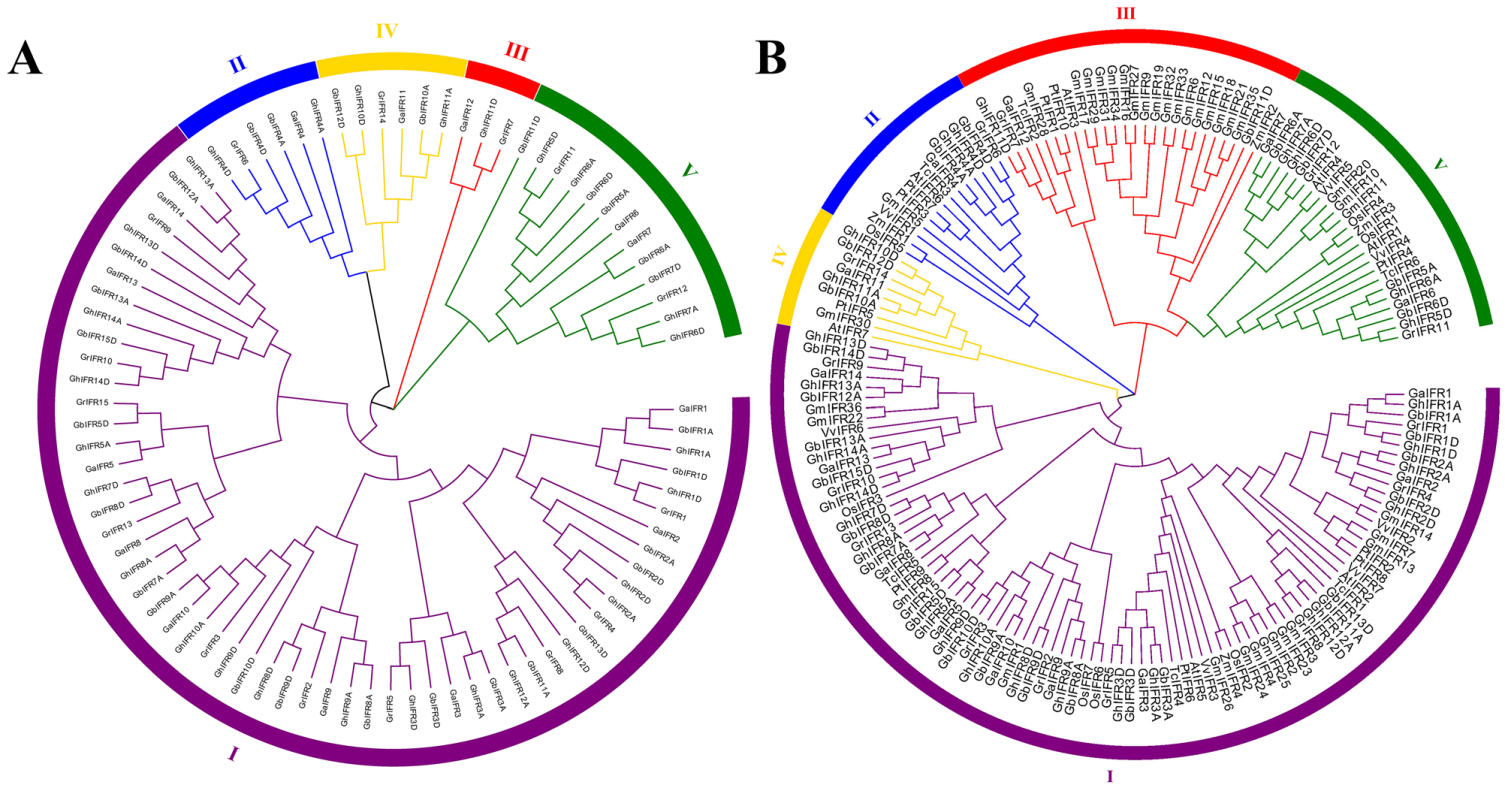
Phylogenetic trees of four *Gossypium* species and other 7 species. (**A**) Phylogenetic trees of the *IFR* genes from four *Gossypium* species, (**B**) Phylogenetic relationship of the *IFR* genes from four *Gossypium* species and other 7 species. The 5 clades, designated as I to V, are marked with different colored backgrounds.

To understand the evolutionary relationship of *IFR* gene families, MEGA7 software was used to construct phylogenetic trees of four cotton species and other 7 species (Fig. 1). According to the branch of evolutionary tree and gene structures, *IFR* genes were divided into 5 clades, among which clade I had the most *IFR* genes, 16 in *G. hirsutum* and 57 in four cotton species. Clade III contained the least *IFR* gene, including 1 in *G. hirsutum* and 3 in four cotton species. In addition, we also found the same trend in the evolutionary tree of four cotton species and seven species. Clade I contained the most *IFR* genes, accounting for 54.94% of the total, with 89 genes. The same clade contained *IFR* genes of four cotton species, and each clade contains *T. cacao*, indicating a close evolutionary relationship between cacao and cotton. In phylogenetic trees, the *GhIFR* gene pairs and *GbIFR* gene pairs were always clustered together, which could be the results of gene duplication. In addition, *IFR* genes in *A. thaliana*, *G. max*, *P. trichocarpa* and four cotton species were all distributed in clade I–V, indicating that *IFR* genes in these plants were evolutionarily linked.

### Chromosomal location of *IFR* gene family

**Figure 2:**
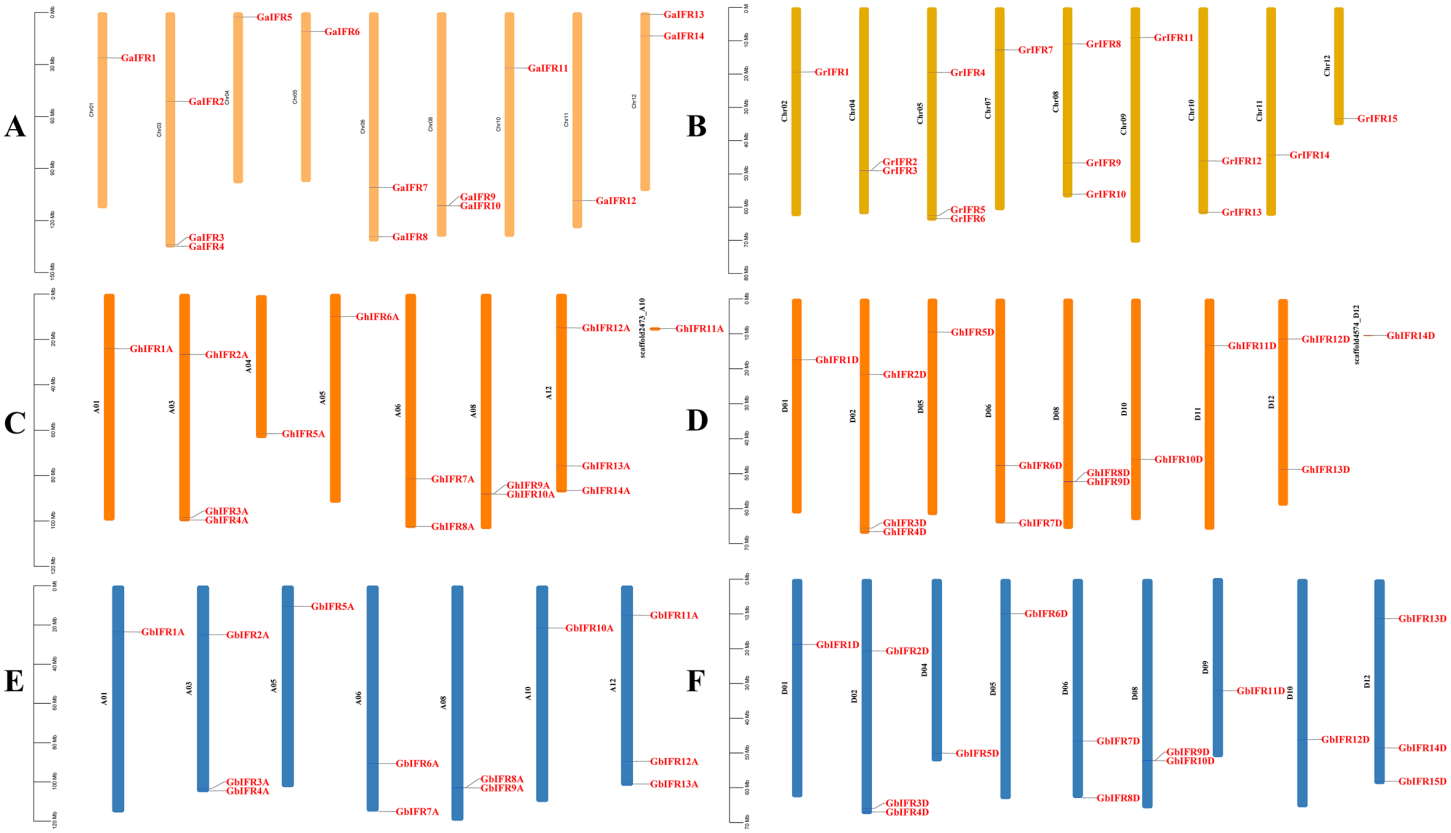
Chromosome distribution map of four *Gossypium* species. (**A**) Chromosomal location of *IFR* genes on chromosomes in *G. arboreum* (Ga), (**B**) Chromosomal location of *IFR* genes on chromosomes in *G. raimondii* (Gr), (**C**) Chromosomal location of *IFR* genes on chromosomes in *G. hirsutum* At sub-genome (GhAt), (**D**) Chromosomal location of *IFR* genes on chromosomes in *G. hirsutum* Dt sub-genome (GhDt), (**E**) Chromosomal location of *IFR* genes on chromosomes in *G. barbadense* At sub-genome (GbAt), (**F**) Chromosomal location of *IFR* genes on chromosomes in *G. barbadense* Dt sub-genome (GbDt). The gene ID on the right side of each chromosome correspond to the approximate locations of each *IFR* gene. The scale of the genome size was given on the left.

To further study the distribution and evolutionary relationship of *IFR* genes on chromosomes, we mapped all genes onto corresponding chromosomes (Fig. 2). Only two genes were not located on the chromosome (*GhIFR11A* and *GhIFR14D*), and all the other genes were unevenly distributed on their chromosomes. In *G. arboreum*, Chr3 had relatively most *GaIFR* genes, with 3 genes, Chr1, Chr4, Chr5, Chr10, and Chr11 had the smallest number of *IFR* genes, with 1. No genes were distributed on Chr2, Chr7, and Chr9, and no tandem duplication events occurred. In *G. raimondii*, Chr5 and Chr8 had relatively most *GrIFR* genes, including 3 and 3 genes, respectively. No genes were found on Chr1, Chr3, and Chr6. In the At subgroup of *G. hirsutum* and *G. barbadense*, the number of *IFR* genes on Chr3 and Chr12 was the most, with 3 and 3, respectively. Although the number of *IFR* genes in A/D subgenome was similar, the distribution of these genes in chromosomes was not corresponding. However, the situation was different in the Dt subgroup, the most *IFR* genes on Chr2 in *G. hirsutum*, which was 3, and the most *IFR* genes on Chr2 and Chr12 of *G. barbadense*, with 3 (Table 2).

**Table 2:** Chromosome number distribution map of *IFR* genes in four *Gossypium* species.

Chr. No	Ga	Gh-At	Gb-At	Gr	Gh-Dt	Gb-Dt
Chr.1	1	1	1	0	1	1
Chr.2	0	0	0	1	3	3
Chr.3	3	3	3	0	0	0
Chr.4	1	1	0	2	0	1
Chr.5	1	1	1	3	1	1
Chr.6	2	2	2	0	2	2
Chr.7	0	0	0	1	0	0
Chr.8	2	2	2	3	2	2
Chr.9	0	0	0	1	0	1
Chr.10	1	0	1	2	1	1
Chr.11	1	0	0	1	1	0
Chr.12	2	3	3	1	2	3

### Gene duplication and collinearity analysis

**Figure 3:**
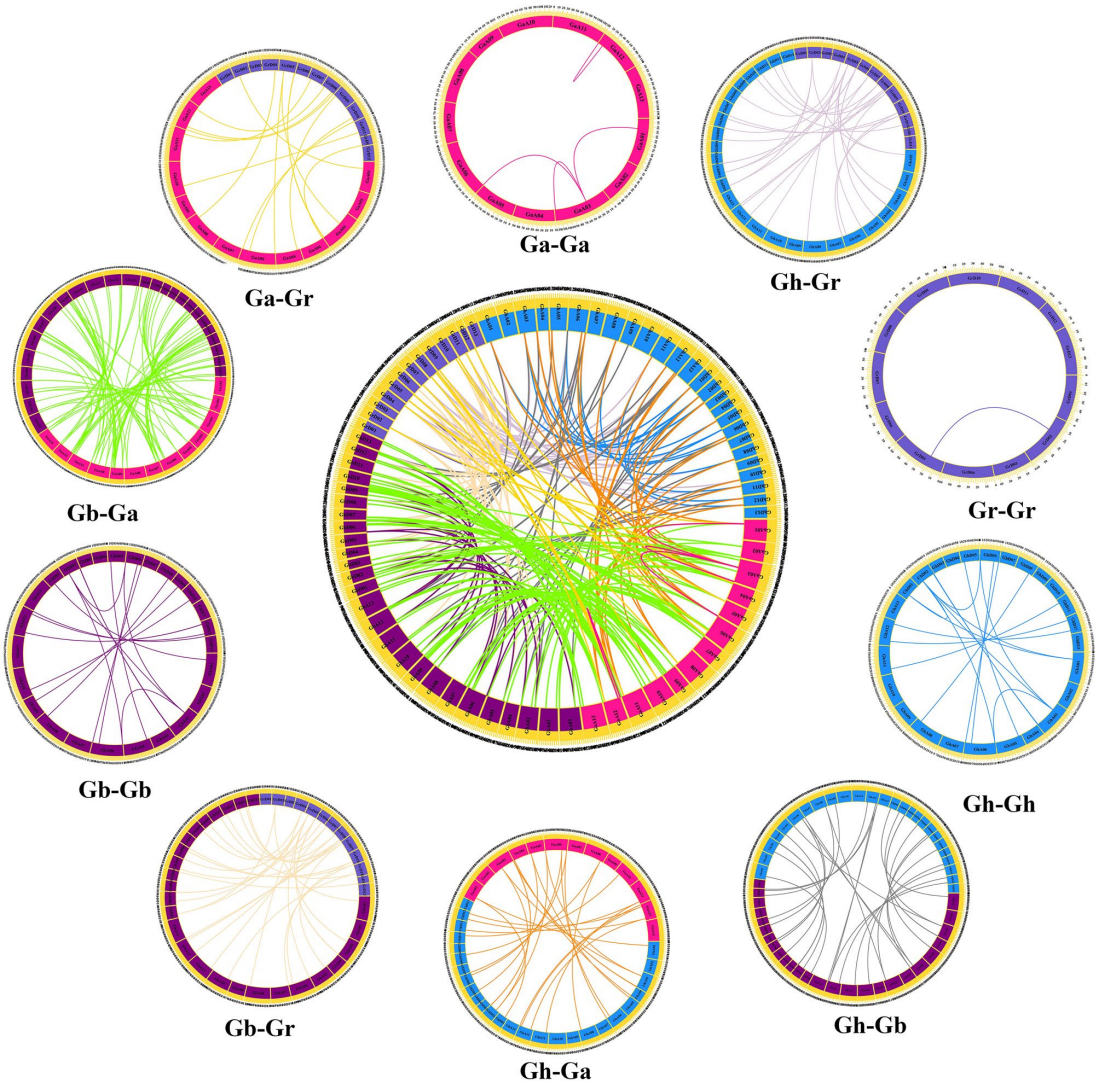
Collinearity analysis of *IFR* duplicated gene pairs in *G. hirsutum*, *G. barbadense*, *G. arboretum*, and *G. raimondii*. Chromosomal lines represented by various colors indicate the syntenic regions around the *IFR* genes.

Gene duplication events are one of the main contributors to evolutionary dynamics, and they play a significant role in the rearrangement and expansion of gene families^20^. Whole genome duplication, segmental duplication, and tandem duplication were the main causes of expansion in plant gene family during the evolution^21^. We identified 267 duplicated gene pairs in 10 combinations (Ga-Ga, Ga-Gb, Ga-Gr, Gb-Gb, Gb-Gr, Gh-Gh, Gh-Ga, Gh-Gb, Gh-Gr and Gr-Gr) (Supplementary Table S2). Among them, 1 gene pair (*GhIFR8D*/*GhIFR9D*) was identified as the tandem duplication, 40 were the segmental duplication and 226 were whole genome duplication (Fig. 3). The number of Lineal/Parallel homologous duplicated gene pairs of *IFR* gene in Gh-Ga, Gh-Gr, Gh-Gb, Gb-Gr, and Gb-Ga was 28, 30, 33, 32, and 93, respectively. The colinear gene pairs of Ga-Ga, Gb-Gb, Gh-Gh and Gr-Gr were 4, 18, 20 and 1, respectively. Therefore, segmental duplication and whole genome duplication were the main driving forces of *IFR* gene family evolution, leading to gene amplification.

### Selection pressure analysis

**Figure 4:**
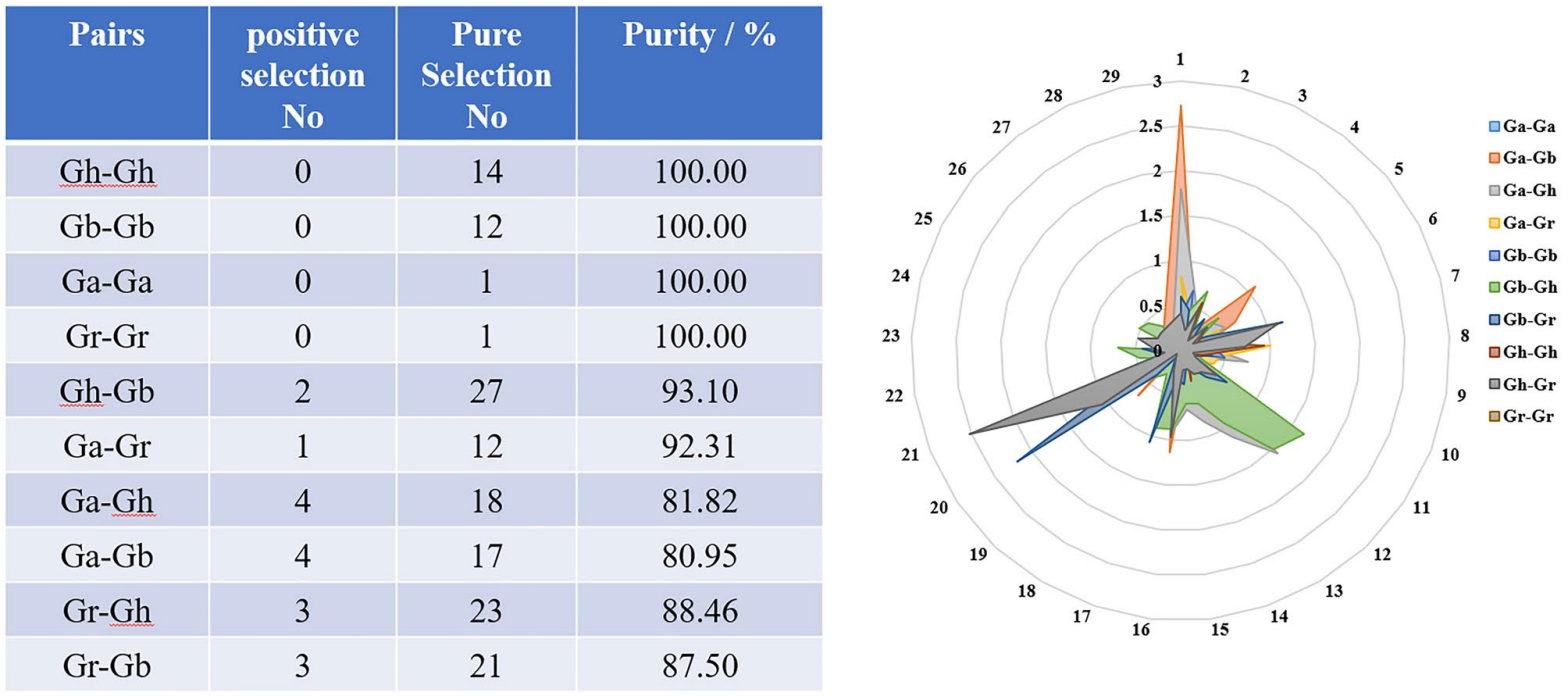
*Ka*/*Ks* ratio analysis of 10 combinations. *Ka* and *Ks* divergence values for (Gh-Gh), (Gb-Gb), (Ga-Ga), (Gr–Gr), (Gh-Gb), (Ga-Gr), (Ga-Gh), (Ga-Gb), (Gr-Gh) and (Gr-Gb) are shown in radar chart. Different colors represent *Ka*/*Ks* gene pairs of 10 groups.

To study the mechanism of *IFR* gene differentiation after polyploid duplication events in cotton, *Ka*, *Ks* and *Ka*/*Ks* of 10 combinations were calculated (Fig. 4, Supplementary Table S3). In general, *Ka* substitution can cause amino acid changes that may alter the conformation and function of proteins, thus causing adaptive changes. *Ka*/*Ks* ratio can determine whether there is selective pressure on the protein-coding gene. The *Ka*/*Ks* ratio was used to infer the selection pressure of duplicated gene pairs. The results showed that 17 duplicated gene pairs with *Ka*/*Ks* > 1, 146 duplicated gene pairs with *Ka*/*Ks* < 1, indicating that most *IFR* genes underwent intense pure selection.

### Analysis of the conserved motifs and gene structures of GhIFR genes

**Figure 5:**
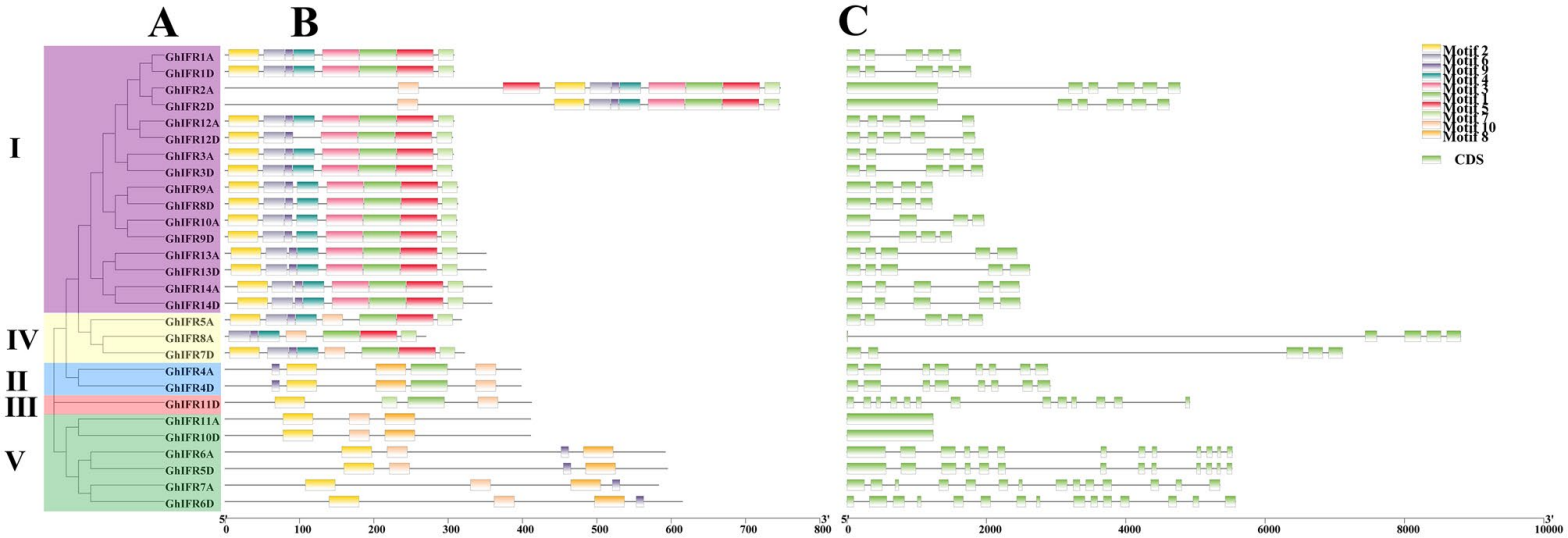
Phylogenetic tree, conserved motifs and gene structure analysis of *GhIFR* genes in *G. hirsutum*. (**A**) Phylogenetic tree of *GhIFR* genes, (**B**) Conserved motifs of *GhIFR* genes, (**C**) Gene structures of *GhIFR* genes. Green boxes indicated exons, and black lines indicated introns.

Diversity of gene structure and differentiation of conserved motifs can promote the evolution of gene families^22^. We analyzed the conserved motifs and gene structures of *GhIFR* genes (Fig. 5). In general, most *IFR* genes belonging to the same clades had similar motif types, arrangements and quantities, but they were not strictly unified in the same clade. For example, each *GhIFR* gene contained 3–10 conserved motifs, especially in subgroups I, II and IV. Except for *GhIFR8A*, all the other *GhIFR* genes contained motif 2, suggesting that motif 2 may be conserved in evolution. In *G. hirsutum*, 2 (7.1%) *IFR* genes had 1 single exon, 5 (17.9%) *IFR* genes had 4 exons, 12 (42.9%) *IFR* genes owned 5 exons and 9 (32.1%) *IFR* gene had more than 5 exons (Fig. 5C).

### Analysis of promoters and heatmap of *GhIFR* genes

**Figure 6:**
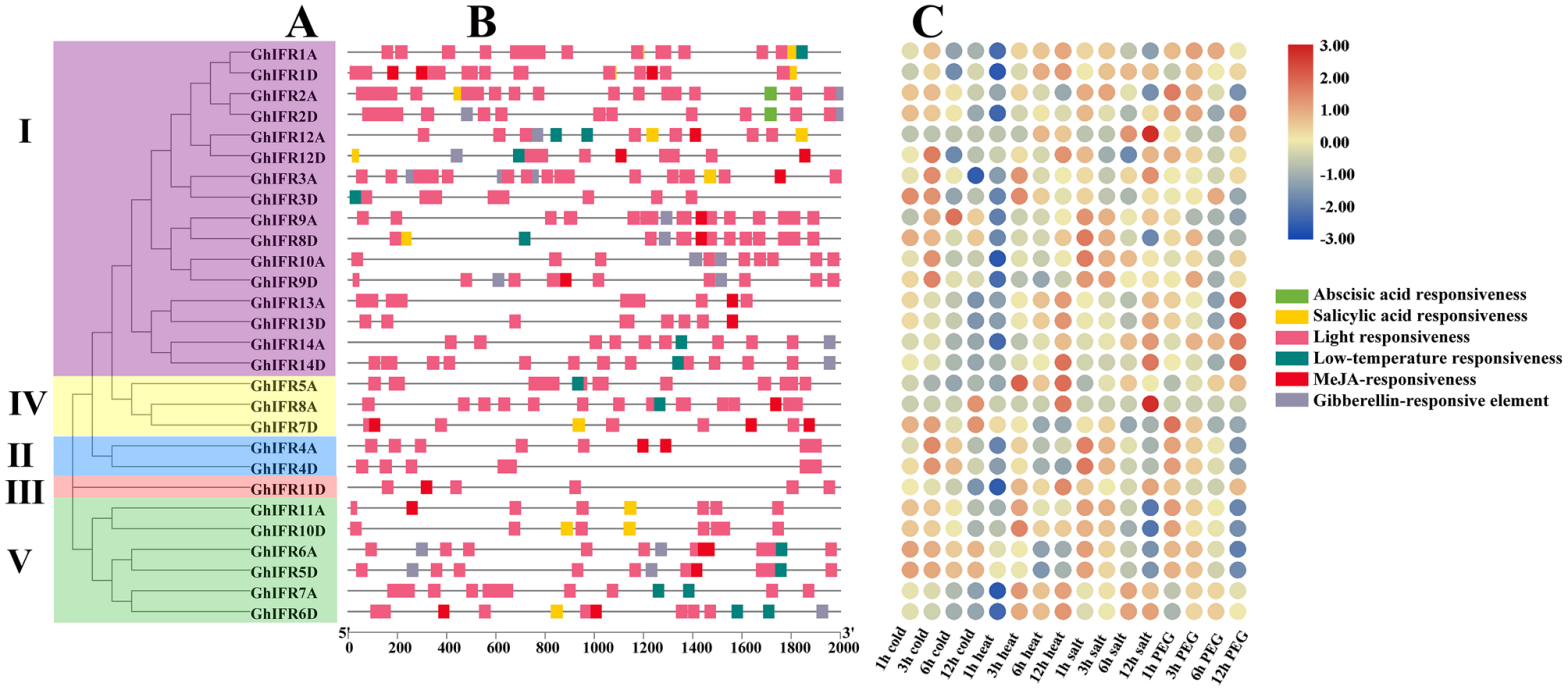
Phylogenetic tree, *cis*-acting elements and heat map analysis of *GhIFR* genes in *G. hirsutum.* (A) Phylogenetic tree of *GhIFR* genes, (B) *Cis*-acting elements in promoters of *GhIFR* genes, (C) Heatmap of *GhIFR* genes under different abiotic stresses.

By analyzing the upstream 2000 bp promoter region of the *GhIFR* genes, we found that most *GhIFRs* contained *cis*-acting elements related to plant hormones and abiotic stresses (Fig. 6B). All *GhIFR* genes contained light responsive elements, suggesting that all *GhIFR* genes may be involved in photosynthesis. 46.4% of the genes contained low-temperature responsive elements, 39.3% of the genes contained *cis*-acting elements responding to salicylic acid. 60.7% of the genes contained *cis*-acting elements related to MeJA. 46.4% of the genes contained gibberellin-responsive *cis*-acting elements. There are more *cis*-acting elements associated with plant hormones than with abiotic stresses. These results suggested that *GhIFR* family genes played an important role in hormone signal transduction and stress response in plants.

To further investigate the potential role of *GhIFR* genes in *G. hirsutum*, we constructed phylogenetic tree and heat maps of *GhIFR* genes under different stresses (cold, heat, salt and drought stress) (Fig. 6A,C). Under cold stress, most *GhIFR* genes tended to be down-regulated in response to cold stress, while most *GhIFR* genes the expression of most *GhIFR* genes was up-regulated under heat, salt and drought stress. The results showed that most *GhIFR* genes responded positively to abiotic stresses.

### qRT-PCR of *GhIFR* genes in response to abiotic stresses

**Figure 7:**
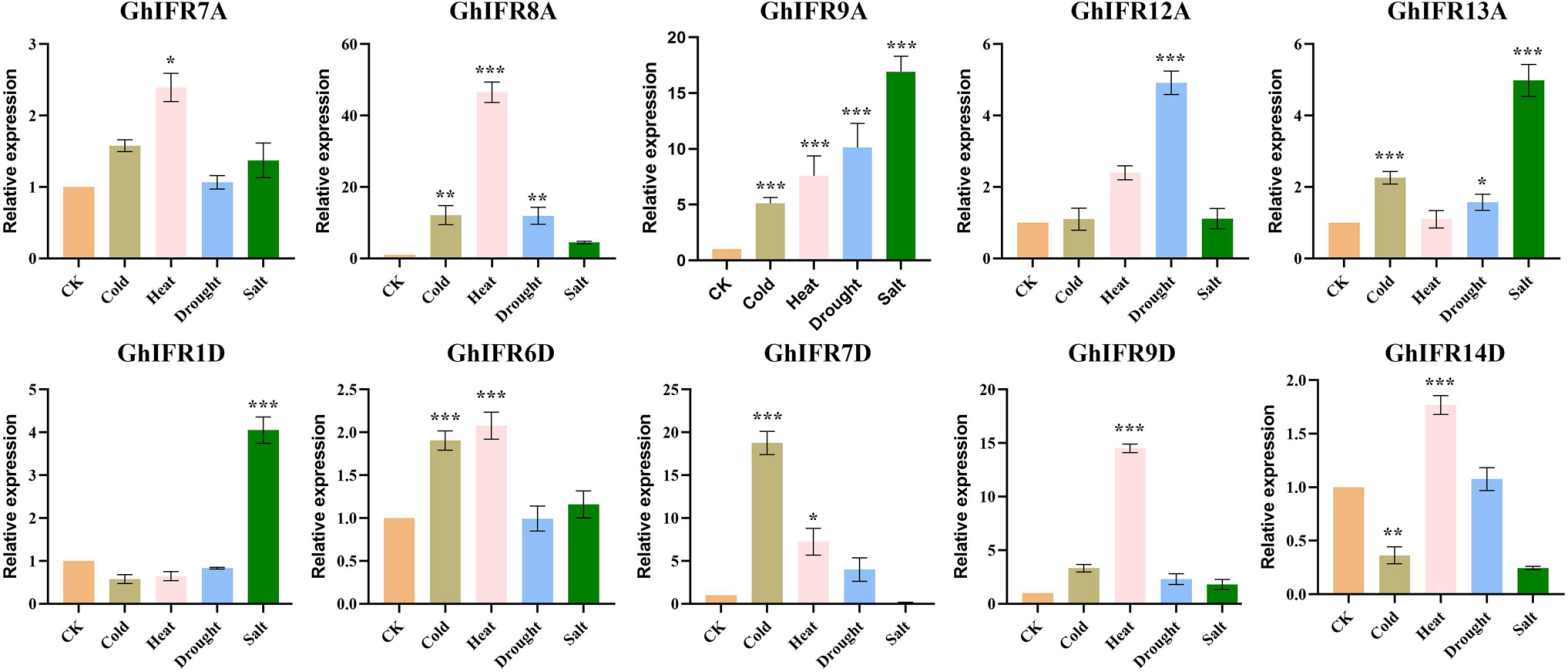
Analysis of the expression patterns of *GhIFR* genes under cold, heat, drought and salt stress by qRT-PCR. The mean values were from three independent biological replicates. Statistical analyses were performed by Student’s t-test (*P < 0.05 and **P < 0.01).

To further confirm the *GhIFR* genes in response to various abiotic stresses, we randomly selected 10 *GhIFR* genes to detect the expression patterns in leaves under cold, heat, drought and salt stress by using qRT-PCR (Fig. 7). The results showed that *GhIFR9A* could be induced by all four different abiotic stresses. Most of *GhIFR* genes responded positively to heat stress, and their expressions were significantly up-regulated, such as *GhIFR7A*, *GhIFR8A*, *GhIFR9A*, *GhIFR6D*, *GhIFR7D*, *GhIFR9D* and *GhIFR14D*. In addition, 6 *GhIFR* genes (*GhIFR8A*, *GhIFR9A*, *GhIFR13A*, *GhIFR6D*, *GhIFR7D* and *GhIFR14D*) were significantly induced under cold stress, showing an upregulation of expression. Two genes, *GhIFR7A* and *GhIFR9D*, were only induced by heat stress, while *GhIFR1D* was only induced by salt stress. These genes that were actively responded to cold, heat, drought, and salt stress could be selected to further verify their functions, such as *GhIFR9A, GhIFR8A* and *GhIFR13A*, especially the *GhIFR9A* gene, which had high expression under four abiotic stresses. Subsequently, the function of the *GhIFR9A* gene will be verified because it is significantly upregulated in various stresses.

### Cotton plants with the *GhIFR9A* silenced by VIGS were sensitive to salt stress

**Figure 8:**
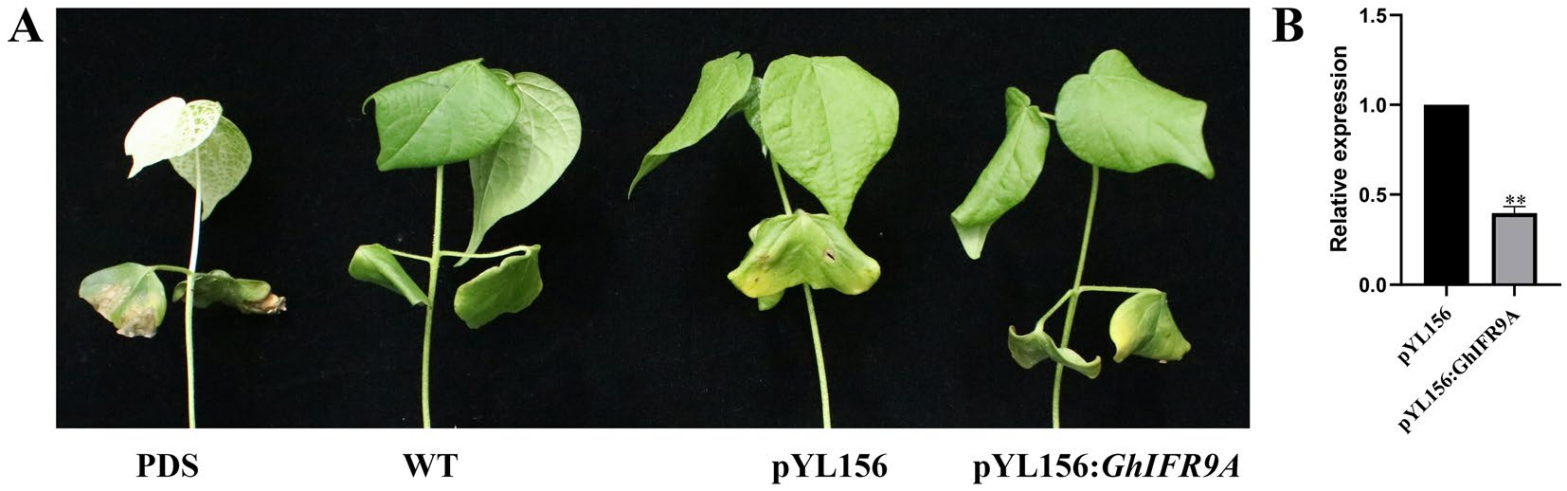
Silencing *GhIFE9A* via VIGS increased sensitivity to salt stress. (**A**) Phenotype of cotton leaves after VIGS, (**B**) qRT-PCR for *GhIFE9A* gene under salt stress. WT: No infection, salt stress: 200 mM NaCl.

According to the results of qRT-PCR, *GhIFR9A* showed a significantly up-regulated trend to the four abiotic stresses, and its expression level was the highest under salt stress. Therefore, we decided to study the function of *GhIFE9A* under salt stress. First, we used VIGS technology to silence this gene, and found that the gene expression was significantly down-regulated after silencing, and under salt stress treatment, cotton seedling exhibited the wilting phenomenon, and pYL156: *GhIFE9A* cotton seedings was more severe than that of pYL156, indicating that *GhIFE9A* was positively regulating the tolerance to salt stress (Fig. 8).

## Discussion

*IFR* gene plays a crucial role in plant growth and development and tolerance to abiotic stress^14^. However, there still lack of systematic understanding of *IFR* gene family in cotton. With the completion of genome sequencing of *G. hirsutum*, *G. barbadense*, *G. arboreum* and *G. raimondii*, it is convenient for us to study the *IFR* gene family. In this study, we identified 28, 28, 14 and 15 *IFR* genes in *G. hirsutum*, *G. barbadense*, *G. arboreum* and *G. raimondii*, respectively. The number of *IFR* genes in two tetraploid cotton is almost the sum of the number of genes in two diploid cottons, which is consistent with the previous study that tetraploid may be formed through hybridization between two subgenomes A and D^23^. In *G. hirsutum*, *IFR* genes in At and Dt subgenome was identical, so we could assume that the translocations and reverse transcript insertion rarely occurred. In addition, 7, 36, 10, 7, 6, 7 and 4 *IFR* genes were identified in *A. thaliana*, *G. max*, *P. trichocarpa*, *O. sativa*, *T. cacao*, *V. vinifera* and *Z. mays*, respectively.

Phylogenetic analysis showed that all species had gene pairs from the same node, indicating that the *IFR* genes in all species had experienced gene duplication that making the expansion of the *IFR* gene family in the process of evolution. Notably, each clade had plants such as *A. thaliana*, *G. max* and *P. trichocarpa*, proving that they were evolutionarily close to cotton. In addition, we found that in every clade, cotton and cacao were on the same evolutionary branch, which was consistent with previous studies that cotton and cocoa came from the same ancestor^18^. The phylogenetic analysis indicated that the *IFR* genes in cotton might have similar biological functions as *TcIFRs*. At the same time, the uneven and random distribution of *IFR* genes suggested that the events of gene loss may occur during the process of evolution, and it might also be due to incomplete genome assembly. Motif is a short sequence of relatively conserved features shared among a group of genes. It may be a recognition sequence or it may encode a functional protein^24^. *GhIFR* genes in the same subfamily had similar gene structures and conserved motifs, which provided support for their clustering in the phylogenetic trees, and the highly conserved sequences in the same subfamily indicated that the *GhIFR* genes may have been duplicated in evolution. All genes contained motif 2, showing that motif 2 might be used to identify the of *IFR* gene family. Almost all genes contain motif 9, except for *GhIFR11A* and *GhIFR11D*, suggesting that these two genes may have lost this motif in evolution.

Gene duplication will lead to functional differentiation of genes, which is necessary for environmental adaptation and speciation^25^. Chromosome distributions and collinearity relationship analysis showed that the amplification of *IFR* genes in cotton was mainly derived from segmental duplication and whole genome duplication. By comparing the number of duplicated gene pairs of Gh-Gr, Gb-Gr, Gh-Ga and Gb-Ga, we found that the number of duplicated gene pairs in Gh-Gr and Gb-Gr was less than that in Gh-Ga and Gb-Ga. These results were consistent with previous results that the A-derived subgenome was more active than the D-derived subgenome during the evolution. There was only one tandem duplication on D08 of *G. hirsutum*, while no tandem duplication in *G. barbadense*, *G. arboreum* and *G. raimondii*, suggesting that there was a special evolutionary pattern in the evolution of different cotton species. In general, the ratio of *Ka*/*Ks* can reflect the evolutionary background of genes. *Ka*/*Ks* = 1 represents neutral selection, and natural selection will not lead to gene mutation. *Ka*/*Ks* > 1 indicates that the gene undergo positive selection, which accelerates the evolution of the gene. *Ka*/*Ks* < 1 indicates that the gene has been purified and selected to eliminate harmful mutations and retain important protein structures^26^. The *Ka*/*Ks* ratio for almost all duplicated gene pairs is less than 1, indicating that the cotton *IFR* gene family has undergone strong pure selection during evolution, with limited functional differentiation after segmental duplication and whole genome duplication.

Isoflavone reductase encoded by *IFR* gene is a class of key enzymes for the synthesis of secondary metabolites such as lignin and isoflavone, and actively responds to biological and abiotic stresses^11,12,27^. In maize, *IFR* gene was activated in response to sulfur starvation^28^. IFR protein was induced by ABA treatment in soybean^9^. Plant hormones play an essential role in plant resistance to abiotic stress. The promoter region of *GhIFR* genes in *G. hirsutum* contained *cis*-acting elements such as light responsive, low temperature responsive and plant hormone responsive. Salicylic acid (SA) could improve abiotic stress tolerance by regulating major metabolic processes in plants^29^. By analyzing *cis*-acting elements of the *GhIFR* genes, we found that 11 of 28 *GhIFR* genes contained SA responsive elements. MeJA is also a plant hormone that may be used against pathogens, salt stress, drought stress, low temperature stress and heavy metal stress^30^, and in this study, 18 (64.3%) *GhIFR* genes contained MeJA-related *cis*-acting elements, especially *GhIFR1D* and *GhIFR7D*, they had 3 MeJA responsive elements, indicating they may respond to adversity stress by regulating the synthesis of MeJA. In conclusion, *GhIFR* genes contained *cis*-acting elements related to abiotic stresses and plant hormone, which suggesting that they may play an important role in cotton growth and development and stress.

Under abiotic stress, plants will produce stress responses and related genes are induced to adapt to various developmental and physiological changes^31^. At the same time, *GhIFR* gene expression pattern analysis under different stresses showed that *GhIFR* gene actively responded to various stresses. It was found that the expression of *IRL* gene in wheat was increased, and the increased expression level was closely related to the synthesis of antioxidants under heat stress^13^. In cotton, we found that most *GhIFR* genes actively responded to heat stress, so we speculated that *IFR* genes may also resist heat stress by increasing the synthesis of antioxidants in cotton, but this needs to be verified in the future.qRT-PCR results showed a key gene, *GhIFR9A*, was significantly up-regulated under cold, heat, salt, and drought stress, which could be selected to further verify its function. There were still some *GhIFR* genes that were not significantly differentially expressed under cold, heat, salt, and drought stress, possibly because they mainly function in other aspects or had lost some functions in evolution. The silence of *GhIFR9A* gene by VIGS experiment showed that the plants that silenced *GhIFR9A* gene were more sensitive to salt stress, which proved that *GhIFR9A* gene could indeed positively regulate the tolerance of salt stress. Previous studies had found that salt stress produced a large amount of reactive oxygen species (ROS)^32^, so we speculated that the *GhIFR9A* gene will eliminate ROS against salt stress through a complex regulatory mechanism.

## Conclusions

In this study, we analyzed the phylogenetic relationship, gene structure, chromosome distribution and *cis*-acting elements of the *IFR* gene family, which greatly enriched our understanding of the cotton *IFR* gene family. In addition, the gene expression profiles under different stress indicated that *GhIFR* genes actively participated in cold, heat, drought and salt stress, which laid a foundation for further analysis of the function of *IFR* genes. *GhIFR9A* gene was induced by salt stress, and silencing *GhIFR9A* gene would cause more severe phenotype in cotton.

## Materials and methods

### Identification of *IFR* gene family members in cotton

To identify the members of the *IFR* gene family in four cotton species (*G. hirsutum*, *G. barbadense*, *G. arboreum* and *G. raimondii*), we used PF05368 (NmrA-like family) in Cotton Functional Genomic Database (CottonFGD) (http://www.cottonfgd.org/) to preliminarily retrieve *IFR* gene family members^33^. The *IFR* genes of other 7 species including *Arabidopsis thaliana* (*A. thaliana*), *Glycine max* (*G. max*), *Populus trichocarpa* (*P. trichocarpa*), *Oryza sativa* (*O. sativa*), *Theobroma cacao* (*T. cacao*), *Vitisvinifera Genoscope* (*V. vinifera*) and *Zea mays* (*Z. mays*) were obtained from the online website Phytozome 13 (https://phytozome-next.jgi.doe.gov/). Then the NCBI CD search website (https://www.ncbi.nlm.nih.gov/Structure/bwrpsb/bwrpsb.cgi) was used to delete C and N terminal to further screen *IFR* family genes^34^. Finally, family genes of four cotton species and 7 species were obtained.

### Phylogenetic tree analysis of *IFR* gene family members

CottonFGD was used to download the protein sequences of four cotton species, and Phytozome 13 was used to obtain the protein sequences of 7 species. MEGA7.0 software was used for multiple sequence alignment. Then, MEGA7.0 software was used to construct the phylogenetic tree of four cotton species using neighbor-joining (NJ) algorithm with 1000 bootstrap repetitions, and the parameter was set as default, and phylogenetic tree of four cotton species and other 7 species was constructed by using Maximum likelihood method with 1000 bootstrap replicates^35^.

### Chromosomal locations of *IFR* genes in cotton

In order to better display the distribution of genes on each chromosome, we used the gff3 file of the genome and gene ID to carry out a visual analysis of the distribution of genes on chromosomes of four cotton species by using TBtools software^36^.

### Duplicated gene pairs and collinearity relationship analysis

The duplicated gene pairs of four *Gossypium* species and the collinearity relationship between different gene pairs was performed using MCScanX^37^. The Advance Circos tool in TBtools was used to make visual analysis of the collinearity and homologous chromosome regions of the four cotton species.

### Calculation of selection pressure

To understand the selection pressure experienced by the *IFR* duplicated gene pairs of four cotton species, the non-synonymous substitution rate (*Ka*), synonymous substitution rate (*Ks*) and *Ka*/*Ks* were calculated to investigate the selection pressure by using TB Tools software.

### Analysis of the conserved motifs and gene structures of *GhIFR* genes in *G. hirsutum*

Multiple Em for Motif Elicitation (MEME, http://meme-suite.org/)^38^ was used to identify the conserved motifs of *GhIFRs*, and the maximum motif number was set to 10, other parameters were set as default. The newick file and gff3 file were then used to visualize the phylogenetic tree, conserved motifs and gene structure using TBtools.

### *Cis*-acting element analysis of *GhIFR* genes in* G. hirsutum*

The upstream 2000 bp sequence was obtained from CottonFGD as the promoter. Then they were submitted to online website PlantCARE (http://bioinformatics.psb.ugent.be/webtools/plantcare/html/)^39^ to get the *cis*-acting elements. TBtools software was used to construct the diagram of phylogenetic tree and *cis*-acting elements of *GhIFR* genes.

### Expression pattern analysis of *GhIFR* genes

RNA-Seq data (PRJNA490626)^40^ including cold, heat, salt and drought stresses was downloaded from NCBI (https://www.ncbi.nlm.nih.gov/). TBtools software was used to visualize the heatmap with FPKM values of *GhIFR* genes.

### qRT-PCR analysis of *GhIFR* genes under different abiotic stresses

To explore the expression patterns of *GhIFR* genes in different abiotic stresses, leaves of cotton exposed to cold (4 °C), heat (40 °C), drought (12% PEG6000) and salt (200 mM NaCl) stress at three leaf stage were collected for RNA extraction at 0 h and 12 h, respectively. Cotton plants treated with ddH_2_O were considered as control, and three biological replicates were taken for each treatment. The total RNA was isolated by using EASYspin Plus plant RNA quick isolation kit (Aidlab Co., LTD, Beijing, China). The pure RNA was reverse-transcribed using TransScript® II one-step gDNA removal and cDNA synthesis supermix (TransGen Biotech Co., LTD, Beijing, China) according to the manufacturer's instructions. Ten *GhIFR* genes were randomly selected for the qRT-PCR experiment, and the primer sequences were shown in Supplementary Table S4. 2^−ΔΔCt^ method was used to measure relative expression levels of *GhIFR* genes^41^.

### Virus‑induced gene silencing (VIGS) experiment

pYL156: *GhIFR9A* vector was constructed with the restriction enzyme cutting site *BamHI* and *SacI*. Primer sequences are shown in Supplementary Table S4. The GV3101 strains carrying pYL156, pYL156: *GhIFR9A*, pYL156: PDS, and pYL192 were cultured and injected into the underside of cotyledons of upland cotton material TM-1. When the plants injected with pYL156: PDS appeared an albino phenotype, it proved that the VIGs experiment was successful. Then the leaves were taken for qRT-PCR experiment. After that, the plants with pYL156, pYL156: *GhIFR9A* were treated with salt stress (200 mM NaCl), and their phenotypes were observed.

## Supplementary Information

Supplementary Tables.

## Data Availability

The datasets used during the current study available from the corresponding author on reasonable request.
