# Supplementary material for: Identification and analysis of isoflavone reductase gene family in Gossypium hirsutum L
Source: Sci Rep. 2023 Apr 7;13:5703. doi: 10.1038/s41598-023-32213-3 (PMC10082034; doi:10.1038/s41598-023-32213-3)
Supplement: Supplementary file 1 — Supplementary Tables. [file 41598_2023_32213_MOESM1_ESM.zip › Supplementary Table S4.docx]

**Supplementary Table S4.** Primer pairs used for qRT-PCR.

| Gene ID | | Primer pairs for qRT-PCR (5'-3') | |
| --- | --- | --- | --- |
| *Actin* | ATCCTCCGTCTTGACCTTG | | TGTCCGTCAGGCAACTCAT |
| *GhIFR7A* | TGGCATTGACCTGAGCAAAC | | ATCAGCTCCAGCAGGTTCTT |
| *GhIFR8A* | GGACAACATTCCGGTGATGG | | GGGTTTAGGAGGGTCAACCA |
| *GhIFR9A* | CCTGCAAGGATGGAGAATGC | | ACAGCCCACCAAGGAAGTAA |
| *GhIFR12A* | CCAGCTGCGAGCATATTCAG | | CCTAGGAGGAACAGTGGCAT |
| *GhIFR13A* | TCCATTGCTTCTTGGCCCTA | | TAAGCTTTGACGCTGCCATC |
| *GhIFR1D* | AAATCCGTCGTGCTGTTGAG | | GGCTGCGACAATGTAGGAAG |
| *GhIFR6D* | TGGCATTGACCTGAGCAAAC | | ATCAGCTCCAGCAGGTTCTT |
| *GhIFR7D* | CGGCTACCTTGGCAAATTCA | | GTTGAGAGGGCGAGTGTAGA |
| *GhIFR9D* | AAATGGGAGACCCTCACTGG | | GTTGGTTAAGCAGCCTTCGT |
| *GhIFR14D* | GGAGAGCATCTTGGACCAGT | | CACCGGTTCTGCCCTATCTA |
| *V-GhIFR9A* | GCCTCCATGGGGATCC ATGGGGAAGAGCAAGGTTCT | | CGAGACGCGTGAGCTCCACAAGCTTGAGTTGCAACA |
